# Re-analysis of SARS-CoV-2-infected host cell proteomics time-course data by impact pathway analysis and network analysis: a potential link with inflammatory response

**DOI:** 10.18632/aging.103524

**Published:** 2020-06-23

**Authors:** Jens-Ole Bock, Ignacio Ortea

**Affiliations:** 1 Cobo Technologies Aps, Maaloev 2760, Denmark; 2 Proteomics Unit, Universidad de Cádiz and Instituto de Investigación e Innovación Biomédica de Cádiz (INiBICA), Cádiz 11002, Spain

**Keywords:** COVID-19, SARS-CoV-2, inflammatory response, proteomics

## Abstract

Coronavirus disease 2019 (COVID-19), caused by an outbreak of the severe acute respiratory syndrome-coronavirus 2 (SARS-CoV-2) in Wuhan, China, has led to an unprecedented health and economic crisis worldwide. To develop treatments that can stop or lessen the symptoms and severity of SARS-CoV-2 infection, it is critical to understand how the virus behaves inside human cells, and so far studies in this area remain scarce. A recent study investigated translatome and proteome host cell changes induced *in vitro* by SARS-CoV-2. Here, we use the publicly available proteomics data from this study to re-analyze the *in vitro* cellular consequences of SARS-CoV-2 infection by impact pathways analysis and network analysis. Notably, proteins linked to the inflammatory response, but also proteins related to chromosome segregation during mitosis, were found to be altered in response to viral infection. Upregulation of inflammatory response proteins is in line with the propagation of inflammatory reaction and lung injury that is observed in advanced stages of COVID-19 patients and which worsens with age.

## INTRODUCTION

Coronavirus disease 2019 (COVID-19), caused by an outbreak of the severe acute respiratory syndrome-coronavirus 2 (SARS-CoV-2) in Wuhan, China, has led to an unprecedented health and economic crisis worldwide [1]. Initially reported in December 2019 in the Chinese city of Wuhan, and potentially linked to a zoonosis related to a wild animal market, COVID-19 has rapidly spread globally, and the World Health Organization (WHO) declared a pandemic on March 11^th^ 2020. As of May 25^th^ 2020, there are 5,304,772 confirmed cases and 342,029 confirmed deaths, with 216 countries affected (WHO, htpps://www.who.int, data accessed on May 25^th^ 2020). These figures make COVID-19 the biggest health emergency of the 21^st^ century. With neither an effective treatment nor vaccine available, the main controlling measure taken by nations has been social distancing followed by partial or total preventative lockdown. These control measures alone have led to the biggest global economic crisis of the 21^st^ century.

COVID-19 typically manifests as an acute respiratory distress syndrome with fever, dry cough and breathing difficulties. Some patients, especially those with specific comorbidities, can rapidly deteriorate and die [2, 3]. A large number of undocumented cases is expected, as a result of asymptomatic carriers and patients with mild symptoms who are not tested for SARS-CoV-2 [4, 5]. The crude global mortality rate is estimated to be 6.5% in WHO reports [6]. However, given the likely high number of undocumented cases it is difficult to calculate the true mortality rate globally and on a nation-wide basis. In any case, COVID-19 spreads at an alarming rate, and both mortality rate and severity increase with age and depend on pre-existing comorbidity, such as hypertension and diabetes, which are age-related diseases. This defines COVID-19 as an aging-dependent disease with outcomes determined by biological age.

To develop treatments that can stop or ameliorate the effects of SARS-CoV-2, we need to understand the biology of the virus and how it behaves inside human cells. This creates an urgent need to decipher the host cell molecular mechanisms that are triggered by viral infection. Cellular factors exploited by SARS-CoV-2 to gain entry into cells have recently been studied, revealing that the virus uses the angiotensin-converting enzyme 2 (ACE2) host cell receptor, together with the serine protease TMPRSS2. On this basis, a TMPRSS2 inhibitor has been proposed as a treatment option [7]. Elsewhere, it has been reported that ACE2 expression is protective against lung injury and that this is downregulated by SARS-CoV-1 [8, 9], which might promote lung injury, therefore worsening the prognosis of the disease, but it has not been demonstrated yet whether SARS-CoV-2 also interferes with ACE2 expression [7].

However, knowledge about what goes on inside human cells after the entrance of SARS-CoV-2 remains scarce. Host cell proteomics studies that measure changes in protein abundance following viral entry and subsequent global pathway and network analysis can shed some light on the mechanisms that are used and/or altered by the virus and may reveal novel drug targets. To the best of our knowledge, the first available study describing translatome and proteome host cell changes induced by SARS-CoV-2 was conducted by Bojkova et al. [10]. Here, the authors used Cytoscape and ReactomeFI to propose overrepresented pathways that could be targeted by potential treatment compounds. In our study, we use the publicly available proteomics data from Bojkova et al. [10] to re-analyze the cellular mechanisms altered upon viral infection by impact pathways analysis [11] and network analysis.

## RESULTS

**Figure 1:**
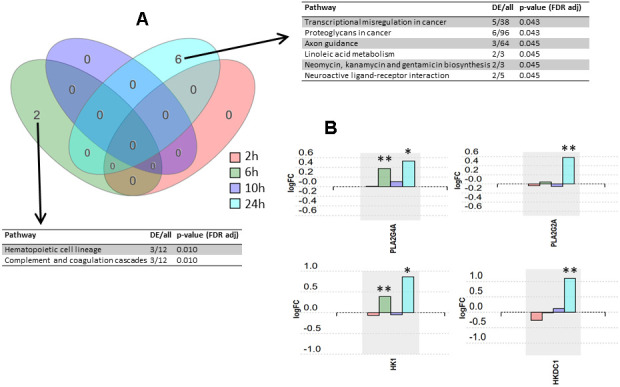
**Pathway analysis results.** (**A**) Venn diagram representing the intersections of pathway sets associated with the four post-infection time points. Pathways were considered significant according to a p-value calculated by iPathway Guide software using a hypergeometric distribution and adjusted using false discovery rate. DE, differentially expressed proteins. (**B**) Expression changes over four post-infection time points for proteins PLA2G4A, PLA2G2A, HK1, and HKDC1. * p-value < 0.05, ** p-value < 0.001.

The input dataset for the analysis, formatted from Bojkova et al. [10], is compiled in Supplementary Table 1. The implicated pathways were analyzed using iPathwayGuide software. Significantly impacted pathways according to our analysis are shown in Figure 1 (see Supplementary Tables 2, 3 for the results from the pathway analysis for the metanalysis and for the 24 h time point, respectively). After false discovery rate (FDR) correction, six pathways were found to be significantly impacted at 24 h post-infection, two pathways were found to be significantly impacted at 6 h post-infection, and no pathways were found to be significantly impacted at 2 h and 10 h post-infection (Figure 1A). Expression changes for selected proteins over post-infection time points are shown in Figure 1B.

**Figure 2:**
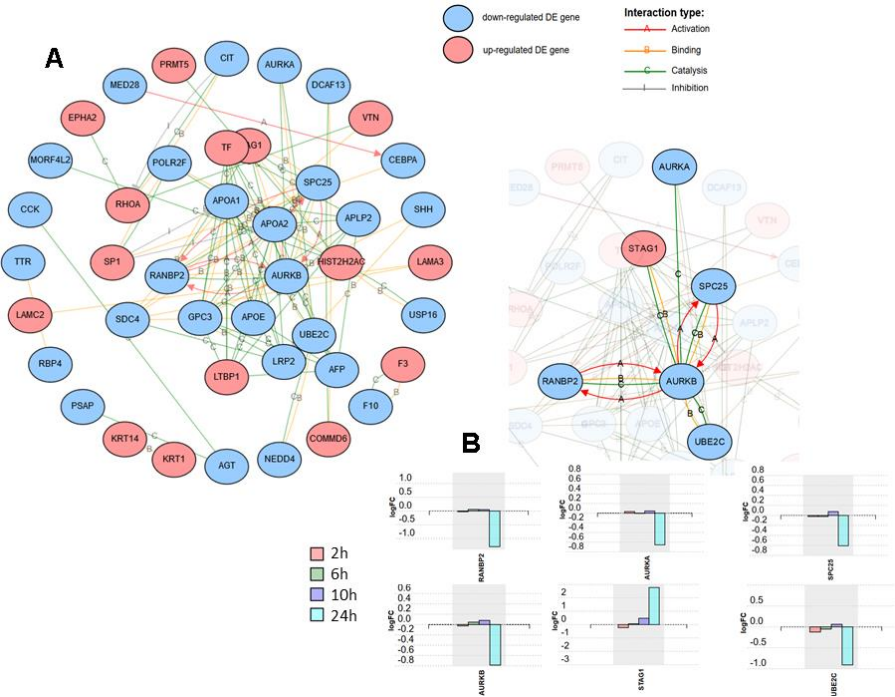
**Network analysis including the 125 differentially expressed proteins at 24 h after SARS-CoV-2 in Caco-2 cells.** Activation, binding, catalysis, and inhibition regulatory interactions are included. (**A**) Network with the isolated nodes hidden. (**B**) Six-protein subnetwork with the interactions for RANBP2, showing the expression changes for each time point for the six proteins.

The differentially expressed proteins at the time point that revealed the most pronounced changes (24 h post-infection), were subjected to network analysis using iPathwayGuide. The interactions included were activation, binding, catalysis, expression, and inhibition. Confidence score for protein-protein interaction was set at 900 (high). The resulting network is shown in Figure 2A. One of the subnetworks with the highest number of interactions, comprised of six proteins, is shown in Figure 2B, together with the expression change profile over post-infection time for these six proteins.

## DISCUSSION

The significantly affected pathways were analyzed using iPathwayGuide software, which implements an ‘impact analysis’ approach, taking into consideration not only the over-representation of differentially expressed genes in a given pathway (i.e. enrichment analysis), but also topological information such as the direction and type of all signals in a pathway, and the position, role, and type of each protein [11]. Although six pathways were found to be significantly impacted at 24 h post-infection, and two at 6 h post-infection, the number of differentially expressed (DE) proteins in these pathways was low (ranging from 2 to 6 proteins). For instance, the pathway ‘transcriptional misregulation in cancer’ had 5 DE proteins out of the 38 proteins included in the pathway, the ‘proteoglycans in cancer’ pathway had 6 DE proteins out of 96 in total in that pathway, and the ‘axon guidance’ pathway had 3 DE proteins out of a total of 64 proteins in that pathway. Thus, we consider the experimental evidence for SARS-CoV-2 having an impact on these mechanisms to be relatively weak. However, while the overall number of DE proteins was low, the ratio of DE proteins to total proteins in three other significant pathways was higher, and these warranted further attention. These three pathways are ‘linoleic acid metabolism’ pathway, ‘neomycin, kanamycin and gentamicin biosynthesis’ pathway, and ‘neuroactive ligand-receptor interaction’ pathway. The linoleic acid metabolism pathway is linked to arachidonic acid metabolism and eicosanoids pathway, and it could therefore play a role in the inflammatory response observed in disease stages II and III in COVID-19 patients [12]. In fact, the two proteins found to be differentially expressed in this pathway at 24 h post-infection, PLA2G4A (cytosolic phospholipase A2) and PLA2G2A (phospholipase A2, membrane associated), are key components of the phospholipase A2 group, which has previously been suggested to participate in a key mechanism of the inflammatory reaction [13]. Additionally, the contribution of the phospholipase A2 group to inflammation and eicosanoid profile in arthritis [14] and in cardiovascular diseases has been demonstrated [15]. When looking at the overall trend in protein expression over the whole time-course, PLA2G4A and PLA2G2A appear to share the same expression profile with a clear increase at 24 h post-infection (Figure 1B). This observation suggests that these two proteins may serve as early systemic biomarkers for COVID-19 infection.

Two proteins from the neomycin, kanamycin and gentamicin biosynthesis pathway were significantly up-regulated at 24 h post-infection (Figure 1B). These are HK1 (hexokinase 1) and HKDC1 (hexokinase domain containing 1), which are proteins related to glucose use and homeostasis [16, 17]. These proteins also belong to the glycolysis/gluconeogenesis pathway, since they participate in the first step of glycolysis where the glucose ring is phosphorylated. The ‘glycolysis/gluconeogenesis’ pathway also appeared in our pathway analysis for the 24 h post-infection time point (although not statistically significant) (Supplementary Table 3), and, according to the nature of the cells used in the assay, it should be a better match than the aminoglycoside antibiotics biosynthesis pathway. Interestingly, HK has previously been associated with the inflammatory response in autoimmune disorders, and deoxy-D-glucose (2-DG), an inhibitor of HK, has been proposed to ameliorate autoimmune inflammation [18]. Recently, 2-DG has been shown to inhibit SARS-CoV-2 replication in Caco-2 cells [10], as well as inhibiting rhinovirus infection and inflammation in a murine model [19]. Given these findings, we believe that a potential link between hexokinase and SARS-CoV-2 infection and the related inflammation response deserves further investigation.

Figure 2A shows the network formed by the DE proteins, excluding isolated nodes. One of the subnetworks with a higher number of connections is the one formed by RANBP2 (E3 SUMO-protein ligase RanBP2) (Figure 2B). RANBP2 forms a complex at the nuclear pore with TRIM5α, a cytoplasmic restriction factor that blocks post-entry retroviral infection and is regulated by SUMO. It has been demonstrated that loss of RANBP2 blocked SUMOylation of TRIM5α, suppressing its anti-retroviral activity [20]. Here, RANBP2 exhibited a statistically significant fold-change (log) of -1.295 at 24 h post-infection, thus the role of RAMBP2-TRIM5α in coronavirus infection deserves further consideration. In the same subnetwork as RANBP2, four other proteins that are interestingly related to cell cycle progression, AURKA, AURKB, SPC25 and STAG1, also deserve further attention (Figure 2B). They all participate in the regulation of chromosome segregation during mitosis [21–24]. Three of these four proteins were found to be down-regulated at 24 h post-infection except STAG1, which was strongly up-regulated. In this subnetwork, closely related to AURKB, and also down-regulated, is UBE2C (Ubiquitin-conjugating enzyme E2 C), which is an essential factor of the anaphase promoting complex/cyclosome (APC/C), a cell cycle-regulated ubiquitin ligase that controls progression through mitosis [25].

**Figure 3:**
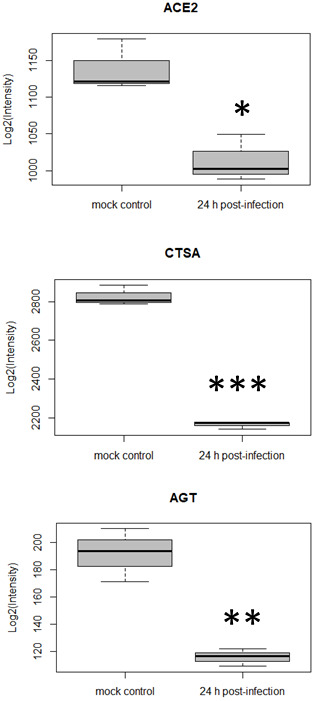
Differential expression for three host cell proteins in the renin-angiotensin system at 24 h post SARS-CoV-2 infection (*p-value <0.05, **p-value <0.01, ***p-value <0.0001, comparison to mock control).

In parallel to re-analyzing the data with alternative tools, we also noticed a trend towards down-regulation of ACE2 over time post-infection (Figure 3). This had not been highlighted by the original authors [10]. In fact, at 24 h post-infection ACE2 presented a fold-change in expression (log) of -0.168 (p-value = 0.01). Coronavirus entry into target cells depends on binding of its spike (S) proteins to a cellular receptor, which facilitates viral attachment to the surface of target cells. ACE2 was reported as the entry receptor for SARS-CoV [26], another coronavirus closely related to SARS-CoV-2, therefore playing a key role in SARS-CoV transmissibility [27]. Similar findings were recently made for ACE2 and SARS-CoV-2 [7]. ACE2 is also a peptidase in the renin-angiotensin system, that converts angiotensin I to angiotensin (1-9) and angiotensin II to angiotensin (1-7), which is a vasodilator. ACE2’s protective role in lung injury is therefore related to its ability to cleave angiotensin II [28, 29]. It was also reported that ACE2 expression protects from lung injury and is downregulated by SARS-CoV [8, 9], which might promote lung injury, therefore worsening the prognosis of the disease. Here, we highlight that SARS-CoV2 also seems to interfere with ACE2 expression, and this may be related to a higher level of lung injury as was demonstrated for SARS-CoV. When inspecting the quantitative data for other proteins in the renin-angiotensin system, two other proteins were found to be down-regulated 24 h post-infection, namely cathepsin A (CTSA) and angiotensinogen (AGT) (Figure 3). We hypothesize that dysregulation of some of the key components of the renin-angiotensin system could be related to the lung injury and worsening observed in COVID-19.

It has also been suggested that differential levels of ACE2 in the cardiac and pulmonary tissues of younger versus older adults may be at least partially responsible for the worse outcomes seen in elderly COVID-19 patients [30]. Elderly people, especially those with hypertension and diabetes, have reduced ACE2 expression and increased levels of angiotensin II proinflammatory signaling. This cohort is therefore potentially more vulnerable to the ACE2 down-regulation that is exasperated by SARS-CoV-2, and we hypothesize that this is one explanation for the exaggerated inflammation and worse outcomes observed in elder populations. The impact of reduced ACE2 expression, together with the poorer clinical outcomes observed when comorbidities are present [31], which is also generally associated to age, makes the link between COVID-19 and aging strong.

ACE inhibitors (ACE-Is) and angiotensin II receptor blockers (ARBs), two common therapies for hypertension, increase ACE2 levels in some tissues such as the myocardium, contributing to protection against cardiovascular disease (CVD) [32]. The observed down-regulation of ACE2 by SARS-CoV-2 could suppress that increase in ACE2 levels mediated by ACE-Is and ARBs, thus counteracting their beneficial effects on hypertension and CVD [32], and explaining the poorer clinical outcome observed when these comorbidities are present. In any case, discontinuation of ACE-Is/ARBs in hypertension patients with COVID-19 might further decrease ACE2 levels, therefore worsening disease prognosis. In support of this notion, a recent study found that COVID-19 patients with untreated hypertension presented higher mortality than those treated with ACE-Is/ARBs [33]. On the other hand, since ACE2 is the receptor for SARS-CoV-2 entry, some authors have speculated about a greater susceptibility to viral infection and disease severity upon the use of ACE-Is and ARBs [34]. However, as of today there is no scientific evidence pointing in that direction, and several scientific societies recommend that hypertension and CVD patients continue their treatments [32].

It is important to note that the use of a colon cell line could be seen as a potential limitation of the study. Superior airways and lungs are the primary targets for SARS-CoV-2, and therefore a primary airway epithelial cell type, such as human bronchial or tracheal epithelial cells (HBEpC/HTEpC) is likely to be a more appropriate model to predict the cell infection profile than the colon cell line used by Bojkova et al. [10]. However, although it was initially thought that SARS-CoV-2 could only infect airway cells, it has since been found to affect numerous tissues and organs, including the intestinal tract [35, 36]. ACE2, the host cell receptor used by SARS-CoV-2 to enter cells, is distributed broadly across human tissues, with similar expression levels in the colon and lung [37, 38]. SARS-CoV-2 has also been found to replicate in gastrointestinal cells *in vivo* and it has been frequently detected in stool, with many patients developing gastrointestinal symptoms [39, 40]. These findings qualify colon cells as an appropriate model for studying SARS-CoV-2 infection and the primary human cell response. On the other hand, Caco-2 cells have been extensively used to study SARS-CoV and are highly permissible for SARS-CoV-2, allowing an analysis of a human model response to viral infection [10, 41, 42].

In summary, this work, through a re-analysis of previous data on the protein expression changes caused by SARS-CoV-2 infection in a cellular model, we point out several proteins related to the inflammatory response and chromosomal segregation that might be modulated by SARS-CoV-2 infection. In the case of proteins related to inflammation, the up-regulation observed could be linked to the propagation of the inflammatory reaction and lung injury that is observed during advanced stages of COVID-19.

## MATERIALS AND METHODS

### Publicly available proteomics data

Proteome measurements from Bojkova et al. [10] were downloaded and used for subsequent analysis. This data consisted of the quantification of 6,381 proteins in human Caco-2 cell secretomes at four time points after infection with SARS-CoV-2 virus. According to Bojkova et al. [10], a TMT-labeling bottom-up quantitative proteomics approach was used to obtain the data, with high pH reverse phase peptide fractionation and mass spectrometry measurement of the peptides using a Thermo QExactive and a nano-liquid chromatography configuration.

### Impact pathway analysis and network analysis

iPathwayGuide (Advaita Corporation, Plymouth, MI, USA) v1910, within the PIPPR pathways analysis framework (COBO Technologies Aps, Maaloev, Denmark), was used to identify significantly impacted pathways and for GO analysis. All quantified proteins were included in the analysis, and the threshold for considering a protein as differentially expressed (DE) was fold-change (log2) higher than 0.5 and p-value below 0.05. Data was analyzed in the context of pathways obtained from the Kyoto Encyclopedia of Genes and Genomes (KEGG) database (Release 90.0+/05-29, May 2019). iPathwayGuide was also used for network analysis, using String v11.0 Jan 2019 and BioGRID v3.5.171 Mar 2019 as data sources. The interactions included were activation, binding, catalysis, expression, and inhibition. The confidence score for protein-protein interaction was set at 900 (high).

### Statistical analysis

For impact pathway analysis, iPathwayGuide software calculated a p-value using a hypergeometric distribution. P-values were adjusted using false discovery rate (FDR).

## Supplementary Material

Supplementary Table 1

Supplementary Table 2

Supplementary Table 3
